# Model-based estimates of sex differences in peak power and fatigue index in track cyclists using directed acyclic graphs, inverse probability of treatment weighting, and Bayesian modeling

**DOI:** 10.3389/fphys.2026.1842270

**Published:** 2026-06-18

**Authors:** Javier Gaviria Chavarro, Miguel Ángel Gómez García, Rosa Nury Zambrano Bermeo

**Affiliations:** 1 Doctoral Program in Applied Sciences, Faculty of Basic Sciences, Universidad Santiago de Cali, Cali, Colombia; 2 Institución Universitaria Escuela Nacional del Deporte, Cali, Colombia; 3 Nursing Program, Faculty of Health, Universidad Santiago de Cali, Cali, Colombia

**Keywords:** muscle fatigue, physical fitness, fatigue, muscle strength, bicycle ergometry test

## Abstract

**Objective:**

The purpose of this study was to estimate sex-related differences in peak power and fatigue index in track cyclists under an explicit causal modeling framework integrating directed acyclic graphs, inverse probability of treatment weighting, and hierarchical Bayesian estimation.

**Design and methods:**

A cross-sectional observational study with a quantitative approach was conducted to estimate adjusted sex-related differences in anaerobic performance indicators in track cyclists. Because sex was non-randomized and not experimentally manipulable, the estimates were interpreted as model-based associations under explicit causal assumptions rather than as definitive interventional causal effects. The sample included 21 cyclists, 15 males and 6 females, aged 14–17 years, with at least 3 years of experience in track cycling. Peak power, mean power, relative power outputs, and fatigue index were assessed using a 30-s Wingate-type test performed on the athletes’ own bicycles mounted on a Tacx NEO 2T Smart electromagnetic resistance system in a controlled velodrome-based setting. The analysis integrated DAG-informed covariate selection, exploratory IPTW diagnostics, sensitivity analyses, and Bayesian estimation with convergence diagnostics.

**Results:**

Male cyclists showed higher peak power (mean [M] = 884.16 W; standard deviation [SD] = 130.76) than female cyclists (M = 626.00 W; SD = 59.93). Similarly, the fatigue index was higher in males (M = 46.23%; SD = 1.21) than in females (M = 44.22%; SD = 0.37). IPTW diagnostics indicated limited covariate balance, particularly for body weight and height, and instability of the unpenalized propensity score model. Sensitivity analyses showed that the sex-related difference in peak power was model-dependent, whereas the fatigue index difference was more consistent across model specifications. Four-chain Bayesian models showed adequate convergence, with R-hat values close to 1.00 and effective sample sizes above 7,700 for the main sex-difference parameters.

**Conclusions:**

These findings suggest that sex-related differences in power generation and fatigue responses may be relevant for individualized training planning. However, given the small sample size and the observational cross-sectional design, the results should be interpreted as exploratory model-based estimates.

## 1 Introduction

Track cycling includes both individual and time trial events (Ferguson et al., 2023). The complexity of the discipline demands technical, tactical, and physical components to perform effectively on the track, where athletes exert force on a mechanical device (Aristizabal et al., 2024; Craig and Norton, 2001). In sports performance, physiological differences between men and women have been well documented, particularly in relation to peak power and muscle fatigue. These differences are partly explained by variations in muscle mass, body composition, hormonal profiles, and neuromuscular characteristics (Nuzzo, 2023; Hunter et al., 2023; Hunter and Senefeld, 2024). However, much of the literature on track cycling is descriptive or correlational and does not explicitly consider the causal structure of the data. This limits the ability to interpret sex-related differences in performance indicators within a transparent causal framework.

The historical context surrounding women in cycling is complex and multifactorial. The limited participation of women in this sport is primarily explained by sociocultural factors, whereby female athletes are frequently perceived as slower, less skilled, and inadequately considered for high-performance sporting contexts (Ayala et al., 2021). Moreover, small female sample sizes are commonly reported in the literature due to lower interest in competitive activities and reduced financial compensation compared with their male counterparts (van Uffelen et al., 2017; Dixon et al., 2017). Consequently, these disparities suggest the existence of sex-related differences that extend beyond physiological determinants to include broader social and structural dimensions.

In high-performance settings, coaches and sport scientists seek evidence that not only describes associations but also helps estimate adjusted differences in power output and fatigue while accounting for relevant anthropometric and training-related characteristics. This type of evidence, often referred to as “real-world evidence,” is typically derived from observational data. When such data are not properly analyzed, results may be biased and fail to yield valid conclusions Tenan and Adams (2024).

Although randomized controlled trials are considered the gold standard for establishing causal relationships, their implementation in high-performance sports is frequently unfeasible due to ethical, logistical, or participant availability constraints. Consequently, well-designed and properly analyzed observational studies can strengthen the interpretation of adjusted associations, especially when an explicit framework of variable relationships is constructed using tools such as directed acyclic graphs (DAGs) (Pearl, 2009; Hernán and Robins, 2020; Nuzzo, 2023). DAGs enable the visualization of causal pathways and help avoid common errors, such as inappropriate adjustment for mediators or colliders, which are critical considerations in studies involving multiple physiological determinants.

Moreover, advances in statistical inference have enabled the integration of propensity score models (e.g., inverse probability of treatment weighting [IPTW]) with Bayesian techniques, producing estimates that not only control for confounding bias but also allow results to be interpreted in probabilistic terms. This non-binary perspective focusing on posterior distributions rather than p-values offers advantages in contexts where sample sizes are small and expected effects are subtle yet practically relevant (Körding and Wolpert, 2006; Imbens and Rubin, 2015; Leonelli, 2025; Nuzzo, 2023).

In response to these challenges, researchers in epidemiology and sports science have proposed approaches based on causal inference and hierarchical Bayesian methods, which combine statistical robustness with greater control over uncertainty. For example, Li et al. (2023), in a paper published in *Philosophical Transactions of the Royal Society A*, highlighted the combined use of propensity scores, causal graphs, and Bayesian models to enhance the validity of observational studies. Furthermore, the application of Bayesian models in sports has shown promising results in other disciplines: a recent study in biathlon demonstrated how Bayesian hierarchical models help explain variability in shooting performance and optimize training strategies (Leonelli, 2025).

Based on this background, the objective of this study was to estimate sex-related differences in peak power and fatigue index in track cyclists under an explicit causal modeling framework integrating directed acyclic graphs, inverse probability of treatment weighting, and Bayesian hierarchical estimation. Rather than claiming causal identification in an experimental sense, this approach aimed to provide model-based adjusted estimates conditional on the assumptions encoded in the DAG, including exchangeability, positivity, correct model specification, and the absence of unmeasured confounding. This approach represents an original contribution to the sports science literature by combining theory-driven covariate selection, confounding adjustment, and probabilistic interpretation of uncertainty, which may support more individualized and evidence-informed training strategies.

## 2 Materials and methods

### 2.1 Study design

A cross-sectional observational study with a quantitative approach was conducted to estimate adjusted sex-related differences in anaerobic performance indicators in track cyclists under a causal modeling framework. Because sex was non-randomized and not experimentally manipulable, the estimates were interpreted as model-based associations structured by explicit causal assumptions rather than as definitive interventional causal effects. The analysis was conducted using probabilistic Bayesian modeling techniques adjusted for theoretically justified covariates, with anthropometric variables interpreted as body-size-related covariates rather than pure confounders.

### 2.2 Context

Data collection was carried out at a high-performance sports center equipped with a certified velodrome. The anaerobic power assessment was performed in a controlled velodrome-based testing environment, not during official competition. Athletes used their own bicycles mounted on a Tacx NEO 2T Smart electromagnetic resistance system, which allowed the standardization of external resistance while maintaining a cycling-specific setup. This setting differed from both traditional laboratory cycle-ergometer testing and real competition; therefore, the resulting power values should be interpreted as controlled test outputs rather than competition-derived peak power values.

### 2.3 Participants

The sample consisted of 21 youth track cyclists (aged 14–17 years), selected through purposive, non-probability sampling. Inclusion criteria required participants to have accumulated at least 3 years of systematic track cycling practice and to be enrolled in structured training programs. Homogeneity in training experience minimized variability attributable to early adaptation phases or novice status.

### 2.4 Variables

The primary independent variable was sex (categorical: male/female). The primary outcome variables were absolute peak power, expressed in watts (W), and fatigue index, expressed as a percentage. Additional descriptive power variables included relative peak power (W/kg), absolute mean power (W), and relative mean power (W/kg), because these indicators are commonly used to interpret sprint cycling performance in relation to body mass and sustained anaerobic output. Covariates included training age, defined as years of systematic track-cycling experience, body weight, height, and arm span. These variables were selected because of their theoretical and empirical relevance to cycling performance and anaerobic power production. However, body weight, height, and arm span may also lie partly on pathways linking sex to performance outcomes. Therefore, models adjusted for these anthropometric variables were interpreted as estimates of sex-related differences conditional on body size rather than as total effects of sex. All variables were treated as continuous quantitative variables, except for sex.

### 2.5 Data source

Primary data were collected directly by the research team under controlled conditions at the sports center. All measurements were digitally recorded and organized into structured databases for subsequent analysis.

### 2.6 Bias control

To mitigate bias and improve transparency in covariate selection, a causal diagram was constructed using directed acyclic graphs to make explicit the assumed relationships among sex, anthropometric characteristics, training age, and performance outcomes. This framework was used to clarify the target estimand of the adjusted models rather than to claim definitive causal identification. Because anthropometric variables may plausibly function as intermediate variables rather than purely as confounders, the adjusted models were interpreted as estimating sex-related differences conditional on body size. IPTW was used as an exploratory procedure to evaluate whether weighting could improve comparability between groups. However, because the study was observational, cross-sectional, small, and sex-imbalanced, the weighted estimates were interpreted cautiously. In small datasets, propensity score models may be sensitive to individual observations, limited covariate overlap, separation, and unstable weights. Therefore, the IPTW-based results were considered complementary diagnostic estimates rather than conclusive evidence of successful causal adjustment.

### 2.7 Sample size

The sample size was small (n = 21), reflecting the restricted accessibility of youth track cyclists with systematic competitive experience. This limitation was considered when selecting the analytical strategy and when interpreting the findings. Although Bayesian modeling allows uncertainty to be represented through posterior distributions and may be useful in small-sample contexts, it does not eliminate problems related to limited statistical information, overfitting, unstable estimates, or insufficient covariate overlap. Therefore, the results were interpreted cautiously, emphasizing the magnitude and uncertainty of the adjusted estimates rather than definitive inferential claims.

### 2.8 Instruments

Anthropometric measurements were taken by an International Society for the Advancement of Kinanthropometry (ISAK) Level 2-certified anthropometrist, following the international standards established by the ISAK (Marfell-Jones et al., 2012). Calibrated and validated instruments were used: a Lufkin tape measure for arm span, a Tanita scale for body weight, and a Seca stadiometer for height.

### 2.9 Experimental procedure

Participants attended the velodrome for a single evaluation session. Anthropometric measurements were taken first, followed by specific anaerobic performance tests. Anaerobic capacity was assessed using the Wingate test, adapted to the competitive context of track cycling. The protocol established by Bar-Or (1987)—widely validated for estimating performance in short-duration, high-intensity efforts—was followed. However, instead of using a traditional laboratory cycle ergometer, the test was performed on the athletes’ own bicycles mounted on a Tacx NEO 2T Smart electromagnetic resistance system (model T2875.60, Garmin Ltd., The Netherlands). This device was used to standardize external resistance and record power-related outputs under controlled conditions. The protocol was conducted in a velodrome-based testing environment rather than during official competition; therefore, the power values should be interpreted as controlled Wingate-type test outputs and not as maximal power values obtained during race conditions.

During the test, participants pedaled for 30 s against a load equivalent to 7.5% of their body mass, as stipulated in the classical protocol. Variables recorded included absolute peak power (W), relative peak power (W/kg), absolute mean power (W), relative mean power (W/kg), and fatigue index (%), using the Tacx system’s native software. Absolute power values were retained because they represent the direct mechanical output of the test, while relative power values were included descriptively to account for differences in body mass. The testing environment was controlled for temperature, lighting, and surface characteristics to ensure reproducibility of the protocol.

Prior to the main test, participants completed a 5-min warm-up at moderate intensity, followed by three activation sprints of 2 s each, with 2 min of passive recovery between sprints. This warm-up protocol was designed to optimize neuromuscular readiness and minimize injury risk without inducing pre-test fatigue. The entire evaluation was conducted in a single session per participant and supervised by specialized personnel.

### 2.10 Data analysis

A DAG was constructed to represent the assumed relationships among variables and to clarify the estimand targeted by the adjusted models. Because body weight, height, and arm span may be influenced by sex and may also affect performance, models including these anthropometric variables were interpreted as estimating sex-related differences conditional on body size. Therefore, these estimates should not be interpreted as total causal effects of sex, but rather as model-based adjusted differences under the assumptions encoded in the DAG. In this study, the primary estimand for the fully adjusted models was not the total sex-related difference in performance. Instead, the estimand was defined as the model-based conditional contrast in expected performance between male and female cyclists at comparable values of the included anthropometric variables, namely body weight, height, and arm span, as well as training age. Because these anthropometric variables may partly mediate the relationship between sex and performance, this estimand should not be interpreted as a natural or controlled direct effect of sex, but as an anthropometry-conditioned association under the specified model.

Propensity scores were initially specified using training age, body weight, height, and arm span. However, the unpenalized logistic regression model was not estimable because of a singular matrix, suggesting separation or insufficient covariate overlap. Therefore, an L2-penalized logistic regression model with standardized covariates was used for diagnostic inspection of propensity score overlap, IPTW weight distribution, and covariate balance. Unstabilized and stabilized IPTW weights were examined. No weight truncation was applied. Covariate balance before and after weighting was evaluated using standardized mean differences. Values below 0.10 were considered indicative of adequate balance, whereas larger values were interpreted as residual imbalance.

Bayesian models were used to estimate posterior distributions of sex-related differences in peak power and fatigue index. Weakly informative priors were specified, with normal priors for group-level means and half-normal priors for residual standard deviations. Sampling was performed using Markov Chain Monte Carlo with four independent chains, 2,000 posterior draws per chain, and 1,000 warm-up iterations. A target acceptance rate of 0.95 was specified to reduce the probability of divergent transitions during sampling. Convergence was evaluated using R-hat, effective sample size, visual inspection of trace plots, and the presence of divergent transitions. Posterior predictive checks were conducted to evaluate whether the fitted models reasonably reproduced the observed sex differences in peak power and fatigue index.

Interpretation of results was based on posterior distributions, using the probability of direction (Pd) to quantify certainty regarding the sign of the sex-related difference, along with 94% highest-density credible intervals (HDIs). All analyses were conducted in Python, using PyMC and ArviZ for estimation, visualization, and diagnostic assessment.

### 2.11 Ethics approval and informed consent

This study was approved by the institutional ethics committee of the Institución Universitaria Escuela Nacional del Deporte, in accordance with the principles outlined in the Declaration of Helsinki. All participants provided assent, and their parents or legal guardians provided written informed consent prior to participation. Data confidentiality and participant safety were maintained throughout all evaluation procedures.

## 3 Results

The analysis began with an exploration of descriptive differences between male cyclists (n = 15) and female cyclists (n = 6) across the main anaerobic performance variables. Male cyclists exhibited higher absolute peak power (M = 884.16 W; SD = 130.76) than female cyclists (M = 626.00 W; SD = 59.93). The same pattern was observed for relative peak power, with males reaching 16.41 ± 1.71 W/kg and females reaching 12.30 ± 0.09 W/kg. Absolute mean power was also higher in males (485.99 ± 72.01 W) than in females (338.33 ± 28.58 W), as was relative mean power (8.69 ± 0.95 W/kg vs. 6.85 ± 0.04 W/kg, respectively). The fatigue index was slightly higher in males (46.23 ± 1.21%) than in females (44.22 ± 0.37%).

IPTW diagnostics were conducted to evaluate the feasibility and stability of weighting in this small and sex-imbalanced sample. The unpenalized logistic propensity score model including training age, body weight, height, and arm span was not estimable because of a singular matrix, suggesting separation or insufficient covariate overlap. In particular, height showed no observed overlap between male and female cyclists, with male values ranging from 1.62 to 1.81 m and female values ranging from 1.54 to 1.56 m.

A penalized logistic regression model was therefore used for diagnostic inspection. The propensity score distribution showed limited overlap between groups, with males showing a median propensity score of 0.94 and females a median propensity score of 0.19. Unstabilized IPTW weights ranged from 1.00 to 1.61, with a mean of 1.16 and standard deviation of 0.16. Stabilized weights ranged from 0.32 to 1.07, with a mean of 0.67 and standard deviation of 0.21. No weight truncation was applied.

Covariate balance remained insufficient after weighting. Standardized mean differences before weighting were 0.55 for training age, 1.26 for body weight, 3.20 for height, and 0.36 for arm span. After IPTW, the corresponding standardized mean differences were 0.40, 1.42, 3.93, and 0.43, respectively. These findings indicated that weighting did not achieve adequate covariate balance, particularly for body weight and height. Therefore, IPTW-based estimates were interpreted as exploratory diagnostic summaries rather than as evidence of successful confounding adjustment.

Using diagnostic penalized IPTW weights, the weighted mean peak power was 881.49 W in males and 626.72 W in females, corresponding to a weighted difference of 254.77 W. For the fatigue index, the weighted mean was 46.25% in males and 44.22% in females, corresponding to a weighted difference of 2.03%. These weighted estimates were directionally consistent with the unweighted descriptive differences but should be interpreted cautiously because the balance diagnostics indicated limited comparability between groups.

Bayesian models were then employed to quantify the magnitude and uncertainty of the sex-related mean differences, directly estimating the posterior distribution of the difference between male and female cyclists.

In the four-chain Bayesian models, the posterior mean sex-related difference in peak power was 258.61 W (posterior SD = 59.22), with a 94% HDI ranging from 146.45 to 370.19 W. The posterior probability that male cyclists had higher peak power than female cyclists was greater than 99.9%. For the fatigue index, the posterior mean sex-related difference was 2.01% (posterior SD = 0.54), with a 94% HDI ranging from 0.95 to 2.99%. The posterior probability of a higher fatigue index in males was 99.95%. These estimates indicate a positive sex-related difference under the specified model, although they should be interpreted as exploratory because of the small sample size and limited covariate overlap.

**Figure 1:**
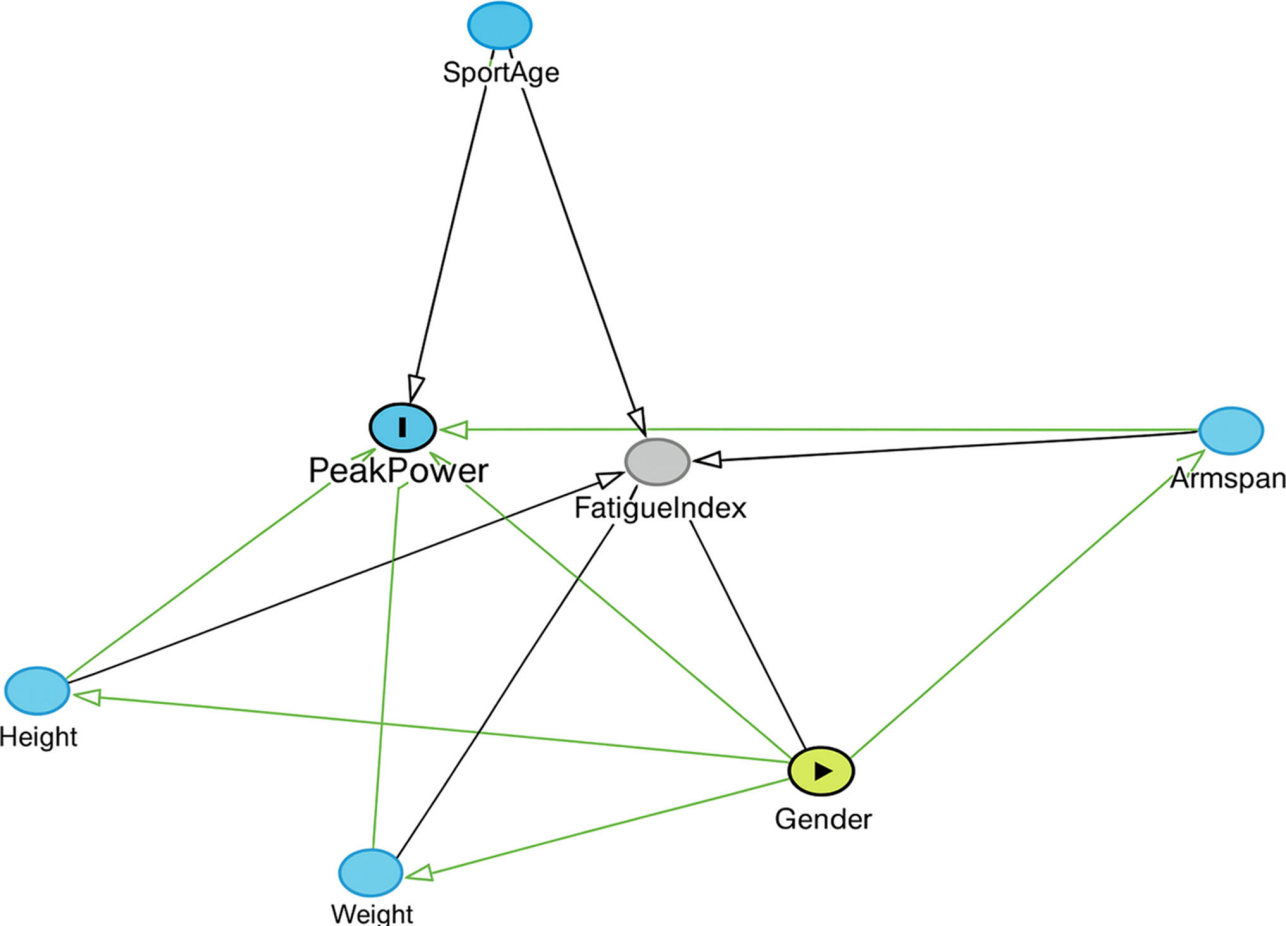
Directed acyclic graph representing the assumed causal structure linking sex, anthropometric characteristics, training age, and neuromuscular performance in track cyclists.

Bayesian diagnostic checks indicated adequate convergence for the monitored parameters. R-hat values were approximately 1.00 for the sex-related difference in both peak power and fatigue index. Effective sample sizes were adequate, with approximate ESS values of 8,000 for the peak power sex difference and 7,750 for the fatigue index sex difference. Posterior predictive checks suggested that the models reasonably reproduced the observed sex differences, with replicated mean differences centered close to the observed differences (**Figure 1**).

Given the small sample size and the potential instability of the IPTW procedure, sensitivity analyses were conducted using simpler linear model specifications. For peak power, the unadjusted model showed a positive sex-related difference of 258.16 W (95% CI: 140.50 to 375.82), and the reduced adjusted model including training age and body weight showed a positive difference of 200.70 W (95% CI: 70.72 to 330.68). However, when height and arm span were additionally included in the full adjusted model, the estimated difference was markedly attenuated and became imprecise (42.23 W; 95% CI: -130.72 to 215.18). This suggests that the peak power estimate was sensitive to model specification and to the inclusion of highly imbalanced anthropometric covariates.

For the fatigue index, the direction of the sex-related difference was more consistent across model specifications. The unadjusted model estimated a difference of 2.02% (95% CI: 0.95 to 3.08), the reduced adjusted model estimated a difference of 2.15% (95% CI: 0.82 to 3.48), and the full adjusted model estimated a difference of 2.89% (95% CI: 0.68 to 5.11). These findings suggest that the fatigue index result was less dependent on model specification than the peak power result, although both outcomes should be interpreted cautiously due to the small sample size and unequal sex distribution.

Directed acyclic graph (DAG) illustrating the hypothesized relationships among sex, anthropometric characteristics, training age, and neuromuscular performance outcomes. Sex was specified as the exposure variable, while peak power and fatigue index were considered performance-related outcomes. Training age was included as a training-related covariate, whereas body weight, height, and arm span were included as performance-related anthropometric covariates. Because anthropometric characteristics may plausibly lie on pathways linking sex to performance outcomes, models adjusted for these variables were interpreted as estimating sex-related differences conditional on body size rather than total causal effects of sex. The diagram does not prove causality but formalizes the assumptions used to guide model specification and interpretation.

## 4 Discussion

The results of this study indicate that, in young track cyclists, male sex was associated with higher peak power and a greater fatigue index relative to female sex in the descriptive and Bayesian sex-difference models. However, because the study was observational, cross-sectional, based on a small and sex-imbalanced sample, and affected by limited covariate overlap, these findings should be interpreted as exploratory model-based estimates obtained under explicit causal assumptions rather than as definitive interventional causal effects. Within this framework, the integration of DAGs, IPTW diagnostics, sensitivity analyses, and Bayesian hierarchical estimation strengthened the transparency of the analytical assumptions and allowed uncertainty to be quantified probabilistically, which is particularly relevant in observational sports research (Abt et al., 2020; Kruschke, 2015).

Therefore, the fully adjusted models answer a narrower descriptive question: among cyclists with similar observed training age and anthropometric characteristics, what is the remaining model-based sex-related difference in peak power or fatigue index? This quantity differs from the total sex-related difference, which includes pathways operating through body size and related anthropometric characteristics. Consequently, attenuation of the peak-power estimate after anthropometric adjustment should be interpreted as evidence of model dependence and pathway conditioning, rather than as evidence that sex has no relationship with power output.

The empirical direction of the findings is consistent with well-established physiological knowledge regarding sex-related differences in anaerobic power and fatigue. Therefore, the main contribution of this study does not lie in identifying a previously unknown physiological pattern, but in illustrating how DAG-informed covariate specification, IPTW diagnostics, sensitivity analyses, and Bayesian estimation can be integrated to produce transparent, uncertainty-aware, and assumption-dependent estimates in a real-world youth track-cycling context.

Several assumptions are necessary for interpreting the adjusted estimates within this analytical framework. Conditional exchangeability assumes that, after adjustment for training age, body weight, height, and arm span, male and female cyclists were comparable with respect to the measured determinants of anaerobic performance (Austin and Stuart, 2015). Positivity assumes sufficient overlap in the distribution of the adjustment variables across sexes, allowing meaningful weighted comparisons (Westreich and Cole, 2010). In addition, the absence of unmeasured confounding assumes that omitted variables, such as maturation status, hormonal profile, training load, nutritional status, event specialization, or neuromuscular history, did not substantially distort the relationship between sex and performance outcomes.

The IPTW diagnostics highlighted important limitations in the comparability of the groups. The unpenalized propensity score model was not estimable because of a singular matrix, suggesting separation or insufficient overlap in the covariate space (Heinze and Schemper, 2002). In particular, height showed no observed overlap between male and female cyclists, weakening the plausibility of the positivity assumption (Westreich and Cole, 2010). Although penalized propensity score diagnostics produced finite weights, covariate balance remained inadequate after weighting, especially for body weight and height (Austin and Stuart, 2015). Accordingly, the IPTW component is best understood as a diagnostic tool for evaluating covariate imbalance rather than as evidence that adjustment successfully removed measured confounding.

The sensitivity analyses further clarified the stability of the findings across model specifications. For peak power, the sex-related difference was evident in the unadjusted and reduced adjusted models, but it was substantially attenuated and became imprecise after simultaneous adjustment for training age, body weight, height, and arm span. This suggests that the peak power estimate is strongly influenced by anthropometric structure and model specification (Martin et al., 2006; Jeukendrup et al., 2000). In contrast, the fatigue index showed a more consistent positive difference across model specifications, indicating a comparatively more stable finding that warrants further investigation in larger samples with better covariate overlap and more balanced sex distributions.

The Bayesian sex-difference model estimated an approximately 259-W higher peak power in males, with a high posterior probability of this difference being positive (Pd > 0.999) (Kruschke, 2015). However, sensitivity analyses showed that this estimate was substantially attenuated after simultaneous adjustment for training age, body weight, height, and arm span, indicating that the peak power result is strongly influenced by anthropometric structure and model specification (Martin et al., 2006). This finding is consistent with previous studies documenting higher power values in men, attributed to greater muscle mass, a higher proportion of fast-twitch type IIx fibers, and increased satellite cell proliferation and protein synthesis. The properties of these fibers favor high contractile velocities through phosphorylation of myosin heavy chains (Hunter and Senefeld, 2024). Endocrine factors such as testosterone have also been shown to play a significant role (Ferguson et al., 2023; Sandbakk et al., 2018). The sex-related difference in power is further linked to torque generation during both dynamic and static contractions (Hunter et al., 2023; Hunter and Senefeld, 2024). Male athletes generally have greater muscle cross-sectional area and longer limb segments, which may facilitate force and power production during cycling, although female athletes may show greater fatigue resistance, partly due to a higher proportion of type I muscle fibers (Joyner et al., 2025; Jeukendrup et al., 2000; Martin et al., 2006). While the relationship between sex and power output has been previously described, this study adds value by estimating adjusted sex-related differences within an explicit causal modeling framework. This approach improves the transparency of the analytical assumptions compared with purely descriptive or unadjusted correlational analyses, although it does not establish causality in an experimental sense.

It should also be noted that the power outputs reported in this study were obtained from a controlled Wingate-type protocol performed on athletes’ own bicycles mounted on an electromagnetic resistance system in a velodrome-based testing environment. Therefore, these values should not be interpreted as direct equivalents of peak power achieved during official competition. Previous cycling research has shown that competition-derived power outputs may differ from controlled testing conditions because of factors such as tactical behavior, pacing, environmental demands, motivation, and race-specific neuromuscular activation. Thus, the present power data are best interpreted as standardized anaerobic test outputs rather than race-specific maximal power outputs.

Ferguson et al. (2023) also highlighted that, although absolute differences in power between sexes are substantial, the slope of the power-duration curve does not differ significantly, suggesting that the disparity is most evident during very short efforts. This observation is consistent with the present findings, as the Wingate test represents a 30-s maximal anaerobic effort.

Regarding the fatigue index, males exhibited a greater percentage decline in performance during the test, with an estimated difference of approximately 2% compared with females (Pd > 0.999) (Beltrami et al., 2023). This aligns with previous evidence showing that men are more susceptible to muscle fatigue in high-intensity exercise, largely explained by a higher rate of glycogen utilization and faster accumulation of metabolites such as inorganic phosphate, dihydrogen phosphate, and hydrogen ions, which acidify pH and impair performance (Barun and Kailashiya, 2015; Hunter, 2004; Ansdell et al., 2020; Gomes et al., 2021). In addition, greater muscle mass contributes to intramuscular blood flow restriction during submaximal efforts, leading to an earlier onset of fatigue (Tiller et al., 2021). Hunter (2004) also reported that women generally exhibit greater resistance to fatigue in submaximal tests due to differences in blood perfusion, muscle metabolism, and neuromuscular efficiency—particularly in the knee extensor muscles—supported by enhanced vasodilation of the femoral artery. This improves tissue oxygenation, enabling more efficient lipid metabolism and glycogen sparing (Ansdell et al., 2020).

These physiological differences may have event-specific implications. For example, women’s greater fatigue resistance could be advantageous in events requiring repeated or prolonged efforts, such as team competitions or multiple qualifying rounds. Recent studies suggest that such sex-specific traits should be taken into account when planning training and recovery strategies for both men and women (Cowley et al., 2021).

From a methodological perspective, this study illustrates both the potential value and the limitations of integrating causal diagrams, IPTW diagnostics, sensitivity analyses, and Bayesian modeling in small real-world sports performance datasets. This emphasis on explicit assumptions, diagnostic reporting, and uncertainty quantification is consistent with broader calls for greater transparency in sport and exercise science research (Borg et al., 2020).

The use of DAGs enabled a transparent and theory-driven selection of adjustment variables, reducing the risk of inappropriate covariate control. IPTW was explored as a strategy to improve covariate balance between groups, but the diagnostic results showed that adequate balance was not achieved in this dataset. However, this procedure does not replace randomization and cannot eliminate bias arising from unmeasured confounding, limited overlap, or model misspecification. Bayesian estimation provided credible intervals (HDIs) and probabilities of direction (Pd), which are increasingly recommended for their interpretability and usefulness in small-sample contexts (van de Schoot et al., 2021).

This approach addresses the limitations of traditional analyses based solely on frequentist hypothesis testing, which are often less informative in small or unbalanced samples. In studies such as this one, characterized by a limited sample size and real-world performance measurements, Bayesian modeling allows uncertainty to be represented more transparently. However, it should not be interpreted as a solution to sparse data, overfitting, limited covariate overlap, or model dependence.

### 4.1 Comparison with similar approaches in sport

The application of hierarchical Bayesian models has recently been promoted in sport as a suitable tool for analyzing time series, comparing small groups, or assessing individual responses to training (Hecksteden et al., 2022; Fonseca et al., 2021). In disciplines such as biathlon, triathlon, and rowing, this approach has been used to estimate probabilities of improvement or decline and to simulate counterfactual training scenarios, highlighting its practical applicability.

From this perspective, the present study should be interpreted primarily as a methodological contribution to the analysis of sex-related performance differences in track cycling. The observed pattern may support individualized monitoring of fatigue, recovery, and training-load responses rather than generalized sex-based prescriptions. For male cyclists, the higher fatigue index observed in this sample suggests that recovery monitoring after high-intensity efforts may be particularly relevant. For female cyclists, the lower fatigue index may indicate comparatively greater resistance to performance decline under the standardized test conditions used in this study. However, these applied interpretations should be considered exploratory and should be confirmed in larger samples with balanced sex distributions and event-specific performance data.

### 4.2 Limitations

The main limitation of this study was the small sample size, which restricts the generalizability of the findings to other populations, age groups, or competitive levels. In addition to the small total sample size, the unequal distribution between male and female cyclists (15 males and 6 females) may have affected the stability of the propensity score model, the IPTW estimates, and the adjusted outcome models. This concern was supported by the IPTW diagnostics: the unpenalized propensity score model was unstable, height showed no observed overlap between groups, and standardized mean differences remained above conventional thresholds after weighting, particularly for body weight and height. Consequently, the positivity assumption may not be fully plausible, and the IPTW results should be interpreted as exploratory diagnostic summaries rather than robust adjusted causal estimates. In addition, the cross-sectional observational design limits causal interpretation, particularly because sex is a non-manipulable exposure and temporality cannot be established in the same way as in longitudinal or experimental designs. Although DAGs and IPTW were used to formalize assumptions and reduce measured confounding, the validity of the adjusted estimates depends on exchangeability, positivity, correct model specification, and the absence of unmeasured confounding.

Variables such as biological maturation, hormonal status, menstrual-cycle phase, training load, nutritional status, cycling-event specialization, neuromuscular history, motivation, psychological stress, and stress-related biomarkers such as cortisol were not included and may have influenced the observed differences (Halson, 2014; Rohleder et al., 2007). This omission is relevant because activation of the hypothalamic–pituitary–adrenal axis may affect glucose availability, perceived exertion, neuromuscular activation, and force production during maximal or supramaximal efforts (Duclos et al., 2003). Therefore, future studies should consider including psychological readiness, motivation scales, perceived-stress indicators, and endocrine markers when specifying causal models of anaerobic performance. Accordingly, the findings should be interpreted as adjusted model-based estimates under explicit causal assumptions rather than definitive causal effects. Sensitivity analyses showed that the estimated sex-related difference in peak power was not fully stable across model specifications, particularly after simultaneous adjustment for training age, body weight, height, and arm span. In contrast, the fatigue index showed a more consistent direction across simpler and fully adjusted models. This pattern suggests that the peak power result should be interpreted with greater caution, whereas the fatigue-index finding appears comparatively more robust, although still exploratory. Although the four-chain Bayesian models showed adequate convergence diagnostics, including R-hat values close to 1.00 and adequate effective sample sizes, Bayesian estimation does not eliminate limitations arising from the small sample size, sex imbalance, limited covariate overlap, observational design, omitted variables, or model dependence.

## 5 Conclusions

The findings of this study suggest the existence of sex-related differences in key anaerobic performance variables in young track cyclists, although their stability differed across outcomes and model specifications. Under the specified causal modeling framework, male athletes showed higher peak power during the 30-s maximal effort and a higher fatigue index than their female counterparts. However, given the observational and cross-sectional design, these results should be interpreted as adjusted model-based estimates rather than definitive interventional causal effects.

From an applied perspective, these findings suggest that sex-related differences in standardized anaerobic test outputs may be considered when individualizing training programs, particularly with regard to power generation capacity, tolerance to repeated efforts, and recovery monitoring. For male cyclists, targeted strategies for monitoring muscle fatigue and implementing active recovery may be considered, given the greater performance decline observed in this sample following high-intensity efforts. In contrast, female cyclists’ lower fatigue index may inform individualized monitoring of training-volume distribution and recovery responses.

Overall, this study contributes mainly by demonstrating the usefulness and limitations of combining DAG-informed modeling, IPTW diagnostics, sensitivity analyses, and Bayesian estimation to examine sex-related performance differences in a small real-world sample of youth track cyclists. The physiological findings are consistent with previous literature and should be interpreted as exploratory, assumption-dependent estimates rather than definitive evidence of a novel physiological pattern.

### 5.1 Practical implications

- In this controlled Wingate-type protocol, male cyclists produced higher absolute and relative peak power than female cyclists.
- Female cyclists showed a lower fatigue index during the controlled test, suggesting a smaller relative decline in performance under these standardized conditions.
- The observed fatigue index difference was relatively consistent across model specifications, whereas the peak power difference was more sensitive to anthropometric adjustment.
- These findings highlight sex-related physiological differences that may influence how male and female athletes respond to intense training and competition.
- These practical implications should be interpreted cautiously because they are based on a small, sex-imbalanced sample and model-dependent estimates. Therefore, they should be used to guide hypothesis generation and individualized monitoring rather than definitive sex-based prescriptions.

## Data Availability

The raw data supporting the conclusions of this article will be made available by the authors, without undue reservation.
